# The Cribriform Plate: A Multifaceted Neuroimmune Hub in CNS Health and Disease

**DOI:** 10.3390/medicina62061125

**Published:** 2026-06-09

**Authors:** Kadır Cetınkaya, Oktay Algın

**Affiliations:** 1 Neurosurgery Department, Hitit Unıversıty Corum Erol Olcok Training and Research Hospital, 19000 Corum, Türkiye; 2 Department of Radiology, Medical Faculty, Ankara University, 06230 Ankara, Türkiye; 3 Interventional MR Clinical R&D Institute, Ankara University, 06100 Ankara, Türkiye; 4 National MR Research Center, Bilkent University, 06800 Ankara, Türkiye

**Keywords:** cribriform plate, glymphatic system, cerebrospinal fluid, lymphatic vessels, neuroimmunology, drug delivery systems

## Abstract

The cribriform plate (CP) functions as a dynamic neuroimmune interface through which olfactory nerve bundles exit the brain within a specialized perineural microenvironment (cpPME). While traditionally viewed as a passive structural barrier, emerging evidence positions the CP as a central hub for cerebrospinal fluid (CSF) drainage, glymphatic–lymphatic clearance, and antigen presentation. This review provides a comprehensive understanding of recent advances in cpPME research, highlighting the adaptive remodeling of the immune landscape in response to neuroinflammation and aging. We critically evaluate the translational gap between rodent models and human physiology, discussing the implications for neurodegenerative diagnostics, neuroinflammatory conditions, infectious diseases and “nose-to-brain” therapeutic delivery. By integrating anatomical, physiological, and immunological perspectives, we offer a comprehensive framework for understanding the CP’s role in CNS homeostasis and its potential as a transformative diagnostic and therapeutic target.

## 1. Introduction

Traditionally, the cribriform plate (CP) has been conceptualized as a mere structural barrier separating the nasal cavity from the intracranial compartment. However, a paradigm shift in neuroimmunology and neurovascular research has unveiled its profound and dynamic role as a critical neuroimmune interface and a central hub for cerebrospinal fluid (CSF) dynamics [1,2]. Emerging evidence positions the CP not merely as a passive conduit but as an active orchestrator of glymphatic–lymphatic clearance, antigen presentation, and immune surveillance within a specialized perineural microenvironment (cpPME) [3,4]. This pathway, which drains into the peripheral lymphatic system, is critically important for the clearance of CNS-derived metabolic waste (glymphatic–lymphatic clearance) and facilitates the exposure of antigens to peripheral immune cells [5,6].

**Figure 1:**
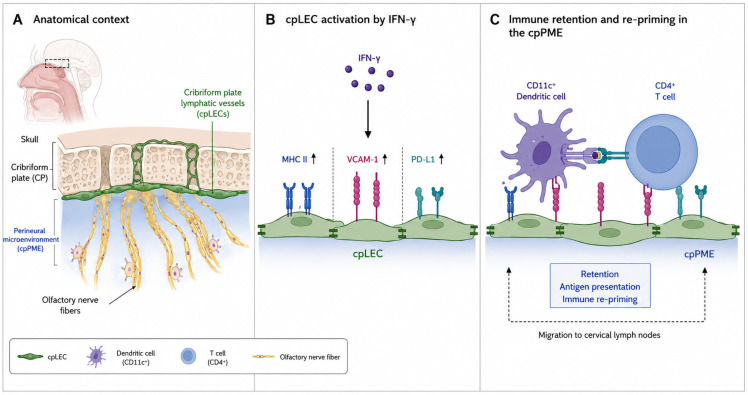
The Cribriform Plate as an Immunoregulatory Niche. (**A**) Anatomical context. Schematic representation of the cribriform plate (CP) region showing olfactory nerve fibers traversing the skull base and their association with cribriform plate lymphatic vessels. These vessels are lined by cribriform plate lymphatic endothelial cells (cpLECs) and are embedded within the cribriform plate perineural microenvironment (cpPME), forming a structural interface between the central nervous system and peripheral immune compartments. (**B**) cpLEC activation during neuroinflammation. Under inflammatory conditions, interferon-γ (IFN-γ), primarily produced by activated T cells and Natural Killer (NK) cells, induces phenotypic activation of cpLECs, characterized by the upregulation of major histocompatibility complex class II (MHC II), vascular cell adhesion molecule-1 (VCAM-1), and programmed death-ligand 1 (PD-L1). This activation leads to an enhanced capacity for antigen presentation and immune cell interaction, facilitating the recruitment and retention of immune cells within the cpPME. The downstream processes involve the sustained activation of immune responses and the efficient clearance of CNS-derived antigens. While the direct role of NK cells within the CP microenvironment is an emerging area, their significant contribution to IFN-γ production in neuroinflammation suggests a potential, yet understudied, involvement that warrants further investigation. (**C**) Immune retention and re-priming within the cpPME. Activated cpLECs facilitate the retention of CD11c^+^ dendritic cells and CD4^+^ T cells within the cpPME. This localized interaction promotes antigen presentation and immune cell re-priming prior to their migration toward cervical lymph nodes, highlighting the cribriform plate as a functional immunoregulatory niche.

Accumulating scientific evidence indicates that lymphatic vessels surrounding the cribriform plate participate both morphologically and functionally in the drainage of CSF and immune cells [7,8]. In particular, during neuroinflammatory conditions (e.g., the experimental autoimmune encephalomyelitis [EAE] model), lymphatic vessels in this region have been shown to expand via lymphangiogenesis, increase their drainage capacity, and undergo phenotypic alterations [9]. This phenotypic transformation is directly associated with the upregulation of genes involved in antigen presentation and immune regulation, including MHC II, VCAM-1, and PD-L1 [9,10]. This “immunoregulatory niche” serves as a critical staging area where CNS-derived signals are translated into systemic immune responses (Figure 1). The relationship between CSF drainage and immune surveillance mediated through the cribriform plate has been proposed as a fundamental mechanism in neuroinflammatory diseases such as multiple sclerosis (MS), meningitis, and stroke, as well as neurodegenerative conditions [3,6]. In neuroinflammatory states, the CP serves as a primary exit for inflammatory debris and activated immune cells, whereas in neurodegenerative diseases, its role is more focused on the clearance of protein aggregates like amyloid-beta and tau.

Although isotope-based and molecular tracer studies (e.g., MR cisternography) in humans suggest CSF efflux toward the cribriform plate and nasal mucosa, recent in vivo studies continue to debate the relative contribution of this pathway to total CSF drainage in humans and its underlying mechanisms [2,11]. While arachnoid granulations (AGs) have long been considered the primary site for CSF absorption into the venous sinuses, recent quantitative studies suggest that the cribriform–lymphatic route may account for up to 50% of total CSF clearance in certain species, particularly under conditions of elevated intracranial pressure (ICP) [2]. In contrast to the passive, pressure-dependent filtration of AGs, the CP route involves active transport through perineural spaces and is influenced by factors such as sleep, head position, and lymphatic contractility [12]. A major point of contention lies in the anatomical scaling between species; while rodents rely heavily on the CP for up to 50% of CSF efflux, the human CP is proportionally smaller, and the relative dominance of dural vs. nasal routes remains to be fully quantified. Nevertheless, the discontinuous structure of the arachnoid barrier over the cribriform plate, together with the reservoir effect created by CSF in this region, supports the hypothesis of its central role in immune interactions [13,14]. It is important to note, however, that current human studies often rely on indirect imaging techniques, which may not fully capture the microanatomical complexity of the cribriform interface compared with high-resolution animal models.

These emerging paradigms highlight the potential significance of the cribriform plate as an “immunoregulatory niche,” where dynamic interactions among CSF drainage, immune surveillance, and the peripheral immune system are orchestrated [14,15]. A comprehensive understanding of the anatomical, physiological, and immunological roles of the cribriform plate may provide new insights into the pathophysiology of neurodegenerative diseases such as Alzheimer’s and Parkinson’s diseases, where inflammation is a commonly raised factor, though its precise role as a cause or consequence remains under investigation [16]. The cribriform plate, as a neuroimmune gateway, offers a unique perspective for analyzing this complex relationship [17,18]. Neuroinflammatory conditions including multiple sclerosis, infectious diseases affecting the CNS, as well as inflammatory disorders of the CNS, including growing interest in atypical parkinsonisms such as Progressive Supranuclear Palsy-Richardson Syndrome (PSP-RS) and Progressive Supranuclear Palsy-Predominant Parkinsonism (PSP-P), where modifying the course of inflammation could be a promising therapeutic strategy. Peripheral inflammatory factors and interleukin-1β (IL-1β) have been linked to cognitive functioning in these conditions, and inflammatory and neurotrophic factors are connected to quality of life [19,20]. This integrative perspective also offers a strong foundation for optimizing intranasal drug delivery strategies and for developing novel diagnostic and therapeutic approaches, including nasal sampling for monitoring neurological diseases [21,22,23]. This review will address the anatomical and microanatomical features of the cribriform plaque, its integration with CSF drainage and the glymphatic system, its role in neuroimmune interactions, and finally, its diagnostic and therapeutic potential.

## 2. Anatomy and Microanatomical Features of the Cribriform Plate

The cribriform plate is a thin, perforated bony structure that forms the horizontal portion of the ethmoid bone and is located at the base of the anterior cranial fossa [24]. This region constitutes an anatomical boundary between the nasal cavity and the inferior surface of the frontal lobe, allowing the passage of olfactory nerve fibers (fila olfactoria) from the nasal mucosa to the olfactory bulb [25]. This unique anatomical organization indicates that the cribriform plate functions not only as a structural barrier but also as a dynamic interface between the central nervous system (CNS) and peripheral structures [4,26].

**Figure 2:**
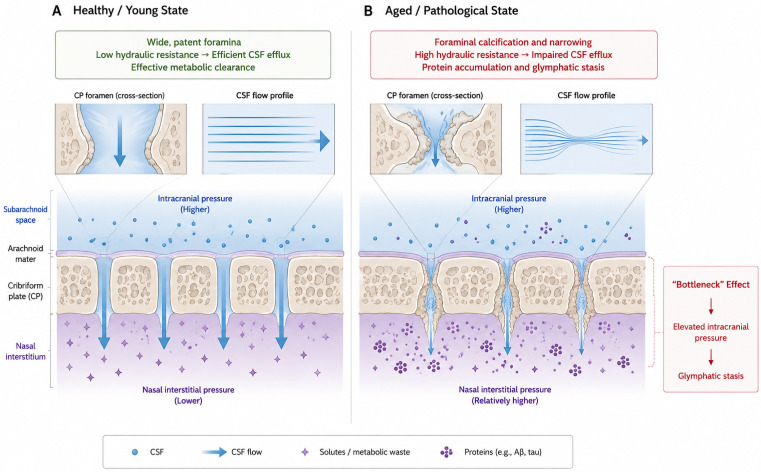
Pressure-Driven CSF Efflux and the “Bottleneck” Effect. This schematic representation illustrates the pressure gradients that drive CSF from the subarachnoid space, through the cribriform plate (CP) foramina, and into the nasal interstitium. The figure provides a comparative analysis between: (**A**) Healthy/Young State: Characterized by wide, patent foramina and low hydraulic resistance, facilitating efficient metabolic clearance. (**B**) Aged/Pathological State: Characterized by foraminal calcification and narrowing, which increases resistance to flow and leads to protein accumulation. The “bottleneck” effect resulting from foraminal narrowing is highlighted as a critical factor in the elevation of intracranial pressure and the development of glymphatic stasis.

Although traditionally considered a passive bony structure, recent experimental studies have demonstrated that the cribriform plate plays an active role in cerebrospinal fluid (CSF) drainage [1,3]. In particular, the perineural spaces surrounding the olfactory nerves have been identified as one of the principal pathways directing CSF toward the nasal submucosa and subsequently into the lymphatic system [27]. Tracer studies in animal models have further confirmed that macromolecules introduced into the CSF rapidly traverse the cribriform plate along olfactory nerve pathways via lymphatic efflux and accumulate within the lymphatic networks of the nasal submucosa [14]. This bulk flow mechanism is driven by the pressure gradient between the subarachnoid space and the nasal interstitium, a process that is highly sensitive to fluctuations in intracranial pressure (ICP) (Figure 2). These findings suggest that, in addition to arachnoid granulations, the cribriform plate serves as a critical alternative pathway for the clearance of CNS-derived waste [28]. This pathway facilitates the passage of small molecules (typically <600 Da), nanocarriers (100–200 nm), and even macromolecules up to 1 μm in experimental models. Such permeability is a critical consideration for the development of intranasal sprays and perineural delivery systems targeting the central nervous system [29,30].

At the microanatomical level, it has been shown that the pia and arachnoid layers extend toward the nasal cavity alongside olfactory nerve fibers, forming “pocket-like” structures in the cribriform plate region [31]. This anatomical arrangement establishes a pathway that facilitates interactions between CSF-borne antigens, soluble molecules, and peripheral immune components [32]. Crucially, the arachnoid barrier in this region is not a continuous seal but a fenestrated layer, allowing for the “leakage” of macromolecules into the perineural microenvironment (cpPME) [33]. Consistent with this, experimental and comparative studies have demonstrated that CSF is directed through the cribriform plate via perineural spaces extending along the olfactory nerves toward the lymphatic structures of the nasal submucosa [34]. Collectively, these findings confirm that the cribriform plate is not merely a structural barrier but also a functional interface between the CNS and the peripheral immune system [35,36].

Aging and pathological processes have been shown to exert significant restrictive effects on CSF drainage pathways [37,38]. In particular, experimental studies indicate that CSF outflow via the nasal/cribriform plate route declines significantly with age [37]. This decline is attributed to both the progressive calcification of the CP foramina and the age-related reduction in the contractility of the collecting lymphatic vessels in the nasal mucosa [39]. Moreover, various comorbidities such as hypertension, diabetes, and cardiovascular diseases can further exacerbate this age-related decline, potentially by affecting vascular integrity and lymphatic function, thereby contributing to impaired CSF clearance [40]. This reduction may contribute to impaired CSF clearance and, consequently, to the progression of age-related neuroinflammatory and neurodegenerative processes [41]. Furthermore, the dynamic role of the cribriform plate as a neuroimmune and clearance interface suggests that its dysfunction may contribute to distinct patterns of neurodegeneration depending on the underlying proteinopathy. Impairment of perineural and glymphatic-associated clearance pathways at this level may differentially influence the accumulation and propagation of misfolded proteins, including tau and α-synuclein, thereby shaping disease-specific pathological trajectories observed in tauopathies and synucleinopathies [42]. However, the interplay between sleep-associated clearance mechanisms and spinal CSF drainage dynamics in the development of these pathological processes remains incompletely understood [43,44]. It is also plausible that the anatomical and functional characteristics of the cribriform plate, including its morphology and the efficiency of CSF drainage, may exhibit variations across different ethnic groups, although further research is needed to fully elucidate these potential differences and their clinical implications.

## 3. Cribriform Plate and CSF Drainage: Integration with the Glymphatic System and Meningeal Lymphatic Network

The glymphatic system is a “hydraulic” clearance pathway in which cerebrospinal fluid (CSF) enters the brain parenchyma via paravascular spaces and mixes with interstitial fluid (ISF) [45]. This system is highly dependent on astrocytic aquaporin-4 (AQP4) water channels, which provide a low-resistance pathway facilitating fluid movement within the interstitial space and, consequently, the clearance of metabolic waste such as β-amyloid [46,47]. The absence or dysfunction of AQP4 channels may reduce waste clearance by approximately 70%, potentially accelerating the pathogenesis of neurodegenerative diseases such as Alzheimer’s disease [45].

The cribriform plate serves as a critical interface for the transition of CSF from the central nervous system to the peripheral lymphatic system. Experimental studies have demonstrated that a substantial proportion of CSF progresses along the olfactory nerve sheaths, drains into the nasal lymphatics in this region, and subsequently reaches the deep cervical lymph nodes [48,49]. The CP acts as the primary “exit port” for the glymphatic–lymphatic axis, where the intraparenchymal waste-laden fluid is finally expelled into the extracranial circulation. This pathway is particularly permeable to soluble macromolecules, with studies indicating efficient transport of molecules up to 150 kDa, making it a promising route for perineural delivery of therapeutic substances to the central nervous system via intranasal sprays [50,51].

In terms of kinetic properties, drainage via the nasal/cribriform plate route in young mice has been shown to be considerably faster and more voluminous than spinal pathways, contributing up to 50% of total CSF efflux in rodents [52,53]. While the human CP is proportionally smaller, its contribution to CSF dynamics is still significant, especially under specific physiological conditions such as sleep [54]. In humans, the sites of CSF outflow are more diverse and complex, involving a delicate balance between traditional venous resorption and emerging lymphatic pathways. While arachnoid granulations have long been considered the primary site for CSF absorption into the dural venous sinuses, recent evidence highlights the significance of “alternative” pathways, including the perineural spaces of cranial nerves and the dural lymphatic network [55]. A critical, yet often overlooked, site of human CSF interaction is the lamina cribrosa (LC) of the optic nerve head [56]. Anatomically, the LC is a sieve-like, fenestrated connective tissue structure that serves as the exit point for retinal ganglion cell axons. It functions as a unique physiological interface where the intraocular pressure from the anterior compartment meets the intracranial pressure within the retrolaminar subarachnoid space [57]. This “blind pouch” of the subarachnoid space allows CSF to exert direct hydrostatic pressure on the posterior surface of the LC. The resulting trans-lamina cribrosa pressure difference (TLCPD) creates a mechanical strain that influences the hydraulic resistance of the optic nerve’s own glymphatic-like clearance system [58]. In humans, this site is particularly vulnerable; a high TLCPD can lead to “glymphatic stasis,” where the impaired drainage of neurotoxic metabolic byproducts, such as amyloid-beta and reactive oxygen species, accumulates around the axons, potentially triggering neurodegenerative cascades seen in glaucoma and normal-tension glaucoma [59]. Thus, the LC is not merely a structural barrier but a dynamic regulatory hub for CSF-mediated metabolic homeostasis at the CNS–ocular interface [59].

During sleep, the enhancement of glymphatic flow is associated with an approximately 60% increase in interstitial space volume within the brain [60]. This expansion reduces tissue resistance and accelerates convective exchange between CSF and ISF [61]. In contrast, during wakefulness, norepinephrine release narrows the interstitial space and suppresses glymphatic flow; thus, the restorative function of sleep is largely attributed to the clearance of neurotoxic waste accumulated throughout the day via these expanded pathways [60,62]. The CP must therefore accommodate significant nocturnal surges in fluid volume, making its patency a prerequisite for the cognitive benefits of sleep.

Aging significantly impairs the efficiency of this drainage network. Evidence suggests that while spinal drainage is relatively preserved, CSF clearance via the cribriform plate is markedly reduced [37]. This reduction hinders the removal of neurotoxic proteins such as β-amyloid and tau from the brain [63,64]. Furthermore, age-related structural deterioration of meningeal lymphatic vessels, along with reduced responsiveness to growth factors such as VEGF-C, further compromises glymphatic function [65]. Collectively, these alterations are considered key contributors to cognitive decline and the development of Alzheimer’s disease [66,67].

## 4. Cribriform Plate and Neuroimmune Interactions: Antigen Drainage and Immunoregulatory Role

The cribriform plate region functions not merely as a passive conduit but as an active neuroimmune “hub” linking the central nervous system with the peripheral immune system [33]. The types of transport through the cribriform plate include bulk flow of CSF along perineural sheaths, transcellular transport across endothelial cells, and paracellular diffusion through fenestrated barriers, all contributing to the dynamic exchange between the CNS and peripheral systems.

### 4.1. cpLEC Phenotypic Transformation

Lymphatic endothelial cells located near the cribriform plate (cpLECs) exhibit unique access to the CSF compartment through discontinuities in the arachnoid barrier [9]. During neuroinflammatory conditions, these cells undergo phenotypic transformation via an IFN-γ-dependent mechanism [68]. Specifically, IFN-γ signaling in cpLECs initiates a JAK-STAT signaling cascade, leading to the upregulation of key immunoregulatory molecules. This cascade promotes the recruitment of immune cells and profoundly modulates the local microenvironment, enhancing the cpLECs’ capacity for antigen presentation and immune cell interaction. The migration of immune cells through the cribriform plate is not a passive process but is governed by highly regulated molecular mechanisms of chemotaxis [9]. Central to this recruitment is the CCR7-CCL21/CCL19 signaling axis. Lymphatic endothelial cells (LECs) within the cribriform plate perineural microenvironment (cpPME) constitutively express or upregulate the chemokines CCL21 and CCL19, particularly under neuroinflammatory conditions. These chemokines bind to the C-C chemokine receptor 7 (CCR7) expressed on the surface of mature dendritic cells (DCs) and T cells. This binding triggers a G-protein-coupled receptor (GPCR) signaling cascade, involving the activation of Phosphoinositide 3-kinase (PI3K) and the recruitment of β-arrestin, which leads to cytoskeletal remodeling and directional cell movement [69]. This molecular “compass” creates a chemotactic gradient that guides CNS-derived antigens and immune cells from the subarachnoid space, through the CP foramina, and into the afferent lymphatic vessels draining toward the cervical lymph nodes. This pathway ensures that the peripheral immune system is continuously informed of the CNS’s immunological status, facilitating either immune tolerance or the initiation of a targeted inflammatory response. In this context, cpLECs upregulate immunoregulatory molecules such as MHC II, VCAM-1, and PD-L1, thereby enhancing their capacity to bind and retain CD11c^+^ dendritic cells and CD4^+^ T cells [9,33]. Notably, the upregulation of MHC II suggests that cpLECs may function as non-professional antigen-presenting cells, directly influencing T-cell activation or tolerance within this niche (Figure 2). When PD-L1 on the surface of cpLECs interacts with PD-1 on the surface of T cells, it delivers an inhibitory signal that can lead to T-cell apoptosis or exhaustion, thereby serving as a critical checkpoint to prevent excessive neuroinflammation and maintain peripheral tolerance.

### 4.2. Lymphangiogenesis and Antigen Trafficking

Inflammatory signals induce VEGFR3-dependent lymphangiogenesis in the vicinity of the cribriform plate [5]. In inflammatory conditions affecting the CNS, such as multiple sclerosis and meningitis, the CP region undergoes significant remodeling characterized by these newly formed lymphatic vessels. These expanded lymphatic networks enhance the drainage of CNS-derived antigens, inflammatory debris, and immune cells to the cervical lymph nodes, thereby playing a pivotal role in the regulation of adaptive immune responses [70]. This adaptive response is crucial for the resolution of inflammation, as it ensures that the peripheral immune system is continuously “updated” on the inflammatory status of the CNS, allowing for a rapid response to infections or autoimmune triggers [12].

## 5. Cribriform Plate Dysfunction in Aging and Neurodegenerative Processes

Aging progressively impairs the structural and functional capacity of clearance pathways associated with the cribriform plate [71].

### 5.1. Structural Deterioration and Obstruction

In elderly individuals and patients with Alzheimer’s disease, the bony apertures of the cribriform plate have been observed to undergo narrowing and increased calcification [72]. This structural alteration mechanically restricts the passage of cerebrospinal fluid (CSF) into the nasal compartment [37]. In addition, aged rats exhibit higher intracranial pressure compared with younger animals (11.52 mmHg vs. 7.04 mmHg), a phenomenon associated with reduced drainage capacity across the cribriform plate [73]. This age-related decline is multifactorial. It involves not only CP foraminal calcification but also reduced lymphatic vessel contractility, loss of astrocytic aquaporin-4 (AQP4) polarization, and structural degradation of the dural lymphatic network. This “foraminal stenosis” creates a bottleneck effect, leading to the accumulation of metabolic byproducts within the olfactory bulb and frontal cortex. While these findings are well established in rodent models, further longitudinal human studies employing advanced high-field magnetic resonance imaging (e.g., 7T or higher) are required to validate the extent of foraminal narrowing and its direct association with cognitive decline in clinical populations.

### 5.2. Protein Accumulation and Clearance Failure

Although CSF drainage via the cribriform plate is significantly faster than spinal pathways in young organisms, this kinetic advantage declines markedly with aging [52]. The accumulation of β-amyloid and tau oligomers may induce glymphatic backflow, resulting in parenchymal deposition and subsequent neuronal injury [74,75]. The failure of the CP–nasal route may thus be a primary driver of the “amyloid cascade” in the early stages of neurodegeneration [76,77].

### 5.3. Viral Factors and the Cribriform Plate

The unique anatomical position of the cribriform plate also makes it a potential gateway for neurotropic viruses, which may exploit the olfactory pathway to access the central nervous system and trigger neuropathological processes associated with neurological diseases [78]. Viruses such as herpes simplex virus, influenza virus, and SARS-CoV-2 may exploit the olfactory nerves and perineural spaces surrounding the cribriform plate to directly access the CNS, thereby bypassing the blood–brain barrier [79]. This direct route of entry highlights the cribriform plate as a critical interface in the context of viral neuropathogenesis and underscores its significance in understanding and preventing virally induced neurological disorders.

## 6. Diagnostic and Therapeutic Perspectives: Nasal Sampling and Drug Delivery

The dynamic interface provided by the cribriform plate offers novel, non-invasive approaches for the management of neurological diseases [80,81,82]. This pathway bypasses the blood–brain barrier (BBB), which remains the primary obstacle for over 98% of small-molecule drugs and nearly 100% of large-molecule therapeutics aimed at the CNS [83].

### 6.1. Nasal Biomarkers

Studies assessing β-amyloid levels in nasal secretions have demonstrated a sensitivity of 65.7% and a specificity of 69.2% for the diagnosis of Alzheimer’s disease [84]. Monitoring oligomeric proteins that reach the nasal mucosa via the cribriform plate represents a promising strategy for early-stage diagnosis [85]. Beyond simple detection, the interaction mechanism here is driven by the pressure-dependent bulk flow of CSF, which “washes” CNS-derived metabolic products into the nasal interstitium. Recent evidence suggests that phosphorylated tau and α-synuclein can also be captured via specialized nasal swabs, reflecting the biochemical state of the brain parenchyma with high sensitivity [86]. This “nasal–glymphatic monitoring” could revolutionize longitudinal tracking of neurodegenerative progression [33]. However, the clinical utility of nasal biomarkers is currently limited by the lack of standardized sampling techniques and the confounding effects of local nasal pathologies, such as chronic rhinosinusitis.

### 6.2. Nose-to-Brain Drug Delivery

To bypass the blood–brain barrier, the olfactory pathways traversing the cribriform plate are utilized as a “backdoor” route [87,88]. The transport across the CP occurs via two primary interconnected mechanisms: the olfactory nerve pathway, and the systemic/lymphatic route. The olfactory pathway involves both slow intracellular axonal transport and rapid extracellular bulk flow within the perineural spaces [89]. For instance, large molecules such as albumin have been shown to reach the brain via this pathway, achieving peak concentrations in the striatum and olfactory bulb within the first 60 min following administration [90,91]. Crucially, the interaction between the olfactory system plays a regulatory role. Trigeminal perivascular spaces offer an additional entry point to the brainstem, and its activation can modulate the permeability of the olfactory barrier through localized neurogenic inflammation [92]. This route enables enhanced central nervous system delivery of therapeutic agents while minimizing systemic toxicity [29]. The use of mucoadhesive nanoparticles and in situ gelling systems can further enhance the residence time of drugs at the CP interface, maximizing their central delivery while minimizing systemic side effects. Modern strategies focus on “mucoadhesive” polymers like chitosan and “tight junction modulators” that facilitate paracellular movement through the fenestrated arachnoid layer. Furthermore, functionalizing nanocarriers with ligands such as lactoferrin can exploit receptor-mediated transcytosis across the olfactory epithelium, maximizing central delivery while minimizing systemic side effects [93].

## 7. **Conclusions**

This review synthesizes recent advances in understanding the cribriform plate as a dynamic neuroimmune gateway, integrating anatomical, physiological, and immunological perspectives. A key strength lies in highlighting its critical role in CSF drainage, glymphatic-lymphatic clearance, and antigen presentation, thereby offering a comprehensive framework for its involvement in CNS homeostasis and disease. However, the current understanding is not without limitations. A significant challenge is the translational gap between rodent models and human physiology, particularly regarding anatomical scaling and the relative contribution of the CP to total CSF drainage. Current human studies often rely on indirect imaging techniques, which may not fully capture the microanatomical complexity. Future research should focus on developing high-resolution in vivo imaging techniques for humans, exploring ethnic variations in CP morphology and function, and conducting longitudinal studies to better understand the dynamic changes in CP function during aging and disease progression. Further investigation into specific molecular mechanisms governing cpLEC activation and their precise role in immune cell trafficking will also be crucial for developing targeted diagnostic and therapeutic strategies. The cribriform plate is a critical “bio-interface” that orchestrates the complex interplay between CNS fluid dynamics and peripheral immune surveillance. Its role in the glymphatic–lymphatic axis makes it a primary determinant of brain waste clearance, while its immunoregulatory plasticity positions it as a key player in neuroinflammatory responses. As we move toward a more integrated view of brain health, the CP emerges as a high-value target for early diagnosis and targeted therapy. Bridging the gap between experimental models and clinical reality will be the next frontier in leveraging this “neuroimmune gateway” for the management of neurodegenerative and neuroinflammatory diseases.

## Data Availability

No new data were created or analyzed in this study.

## Abbreviations

AQP4	Aquaporin-4
Aβ	Amyloid-beta
CNS	Central Nervous System
CP	Cribriform Plate
CpLEC	Cribriform Plate Lymphatic Endothelial Cell
CpPME	Cribriform Plate Perineural Microenvironment
CSF	Cerebrospinal Fluid
EAE	Experimental Autoimmune Encephalomyelitis
IFN-γ	Interferon-gamma
ISF	Interstitial Fluid
MHC II	Major Histocompatibility Complex Class II
MRI	Magnetic Resonance Imaging
PD-L1	Progammed Death-Ligand 1
Vcam-1	Vascular Cell Adhesion Molecule 1
VEGF-C	Vascular Endothelial Growth Factor C
VEGFR3	Vascular Endothelial Growth Factor Receptor 3
